# *LoopTag* FRET Probe System for Multiplex qPCR Detection of *Borrelia* Species

**DOI:** 10.3390/life11111163

**Published:** 2021-10-31

**Authors:** Henning Hanschmann, Stefan Rödiger, Toni Kramer, Katrin Hanschmann, Michael Steidle, Volker Fingerle, Carsten Schmidt, Werner Lehmann, Peter Schierack

**Affiliations:** 1 Attomol GmbH, 03205 Bronkow, Germany; h.hanschmann@attomol.de (H.H.); tonikramer90@aol.de (T.K.); k.hanschmann@attomol.de (K.H.); w.lehmann@attomol.de (W.L.); 2 Institute of Biotechnology, Faculty of Nature and Science, Brandenburg University of Technology Cottbus-Senftenberg, 01968 Senftenberg, Germany; ycschmidty@gmail.com; 3 Faculty of Health Brandenburg, Brandenburg University of Technology Cottbus-Senftenberg, 03046 Senftenberg, Germany; 4 Laborärzte Sindelfingen, 71065 Sindelfingen, Germany; m.steidle@laboraerzte-sifi.de; 5 German National Reference Center for Borrelia, 85764 Oberschleißheim, Germany; Volker.Fingerle@lgl.bayern.de

**Keywords:** *Borrelia*, *B. Burgdorferi* (sensu lato) complex, real-time PCR, Lyme borreliosis, diagnostic

## Abstract

Background: Laboratory diagnosis of Lyme borreliosis refers to some methods with known limitations. Molecular diagnostics using specific nucleic acid probes may overcome some of these limitations. Methods: We describe the novel reporter fluorescence real-time polymerase chain reaction (PCR) probe system *LoopTag* for detection of *Borrelia* species. Advantages of the *LoopTag* system include having cheap conventional fluorescence dyes, easy primer design, no restrictions for PCR product lengths, robustness, high sequence specificity, applicability for multiplex real-time PCRs, melting curve analysis (single nucleotide polymorphism analysis) over a large temperature range, high sensitivity, and easy adaptation of conventional PCRs. Results: Using the *LoopTag* probe system we were able to detect all nine tested European species belonging to the *Borrelia burgdorferi* (sensu lato) complex and differentiated them from relapsing fever *Borrelia* species. As few as 10 copies of *Borrelia* in one PCR reaction were detectable. Conclusion: We established a novel multiplex probe real-time PCR system, designated *LoopTag*, that is simple, robust, and incorporates melting curve analysis for the detection and in the differentiation of European species belonging to the *Borrelia burgdorferi* s.l. complex.

## 1. Introduction

Lyme borreliosis is a multi-system disorder caused by several species of the *Borrelia burgdorferi* (sensu lato) (s.l.) complex. Only in Europe, it is estimated that there are more than 200,000 cases per year, although the number of unreported cases is very likely higher. *Borrelia* are transmitted to humans by bites of infected ticks. The disease primarily affects the skin, joints, and the nervous system [1,2,3,4,5,6]. In Europe the genospecies *B. burgdorferi* sensu stricto (s.s.), *B. afzelii*, *B. bavariensis*, *B. garinii*, *B. lusitaniae*, *B. spielmanii* are assured to be human pathogenic while for the other detected species, namely *B. bissettii*, *B. valaisiana*, and *B. kurtenbachii* human pathogenicity is unclear. However, the most prevalent species in Europe are *B. garinii*, *B. afzelii*, *B. burgdorferi* s.s., and *B. valaisiana* [4,7].

The diagnosis of Lyme borreliosis is based on medical patient history and clinical symptoms. Microbiological analyses are usually based on the indirect detection of *B. burgdorferi* s.l. infection by antibody detection using an enzyme-bound immunosorbent assay (ELISA). Although the ELISA method is widely used, this method has technical limitations due to the assay principle [8] and biological limitations by delaying antibody formation, high dependence on the stage and disease manifestations, cross-reactivity, and high seroprevalence in healthy populations in endemic areas. The later makes it difficult to detect a re-infection by using such type of test. The cultivation of *Borrelia* spirochete is not applied in clinical practice. This is due to long and challenging cultivation, poor sensitivity, and susceptibility to impurities [9,10].

Besides serological testing, quantitative polymerase chain reactions (qPCRs) are widely used detection methods for diagnostic and research purposes [4,7,9,10,11] since PCRs are faster, easier and in many instances more sensitive than cultures [12]. Advantages of qPCR include quantification and differentiation. The target DNA amount in a sample can be determined by observing the change in fluorescence as a function of the number of PCR cycles. From this, the Cq value (quantification cycle) is calculated using various methods. High Cq values are characteristic for samples with little target DNA in the sample. Further details are described in [13]. There are fundamentally two methods of distinguishing the target DNA. These are, on the one hand, probe-based detection, wherein a target DNA is detected in a sequence-specific manner due to the molecular interactions. On the other hand, a target DNA can be distinguished from other sequences due to its melting temperature (Tm). This presupposes that the melting temperature and constant reaction conditions are determined. These basic approaches can also be combined, as described in the following sections. Quantitative diagnostic data are relevant for establishing correlations between *Borrelia* burden and patient symptoms [12,14].

The potential of the PCR for sensitive and fast nucleic acid detection became obvious immediately after its publication by Saiki et al. in 1986 [15] and was soon adapted in laboratory diagnosis of human *Borrelia* [16]. The PCR is a non-quantitative endpoint reaction and is suitable for sensitive detection of DNA. Shortly after the development of the real-time PCR method with which quantitative detection of target DNA molecules is possible in real-time. Basically, the kinetics of the PCR reaction is observed and evaluated in real time. An important milestone for the usability of this real-time PCR technology was the introduction of specific TaqMan probes and intercalating dyes [17,18]. These probes increased the specificity and accuracy of quantitative assays and reduced the risk of cross-contamination compared to conventional PCRs [13,19]. Though qPCR is very sensitive, the detection limit is mainly negatively affected by sampling (leads to a low amount of input DNA), extraction (loss of sample DNA due to the extraction), and reverse transcription for samples starting with RNA instead of DNA [20]. qPCRs are routinely used for numerous applications like gene expression analysis, genotyping, and pathogen detection [13,21,22].

Several probe and primer systems using quenching processes (e.g., Scorpions, Molecular Beacons, or Ampliflour primer) or Foerster Resonance Energy Transfer (FRET, e.g., hybridization probes) for signal generation [23,24,25] have been developed. Probe systems are also applied in other assay platforms such as microarrays and microbead assays [26]. FRET is a physical phenomenon relying on the proximal distance-dependent radiation-less transfer of energy from an excited donor molecule, that initially absorbs energy, to an acceptor dye molecule leading to a measurable increase in emission at the acceptor dye-specific wavelength. This transfer results in a wavelength shift between excitation and emission. FRET partners basically come into close spatial relation when two different probes hybridize in close spatial proximity. The upstream binding probe has a dye-labeled 3${}^{\prime }$-end, and the downstream binding probe a dye-labeled 5${}^{\prime }$-end, with both dyes forming an active FRET pair only when they bind to a common target. The FRET detection signal increases in direct proportion to the formation of specific homogeneous active FRET pairs binding to targets and allows for reliable readouts in assays [27].

An advantage of FRET systems lies in their use for melting curve analysis. Here, hybridized double-stranded DNA bound with dye is heated until its melting point (Tm), where a sudden decrease in measured fluorescence occurs due to dissociation and release of the dyes. If the Tms of several hybridized DNA sequences differ, then multiplex PCRs can be designed: the presence of different targets can be analyzed by melting curve analyzes, although the same FRET pair is used to visualize the amplification [28]. A disadvantage of classical hybridization probe systems is the need for two labeled probes for the detection of one target, which may reduce sensitivity due to a higher number of included oligonucleotides [29]. This factor is an important consideration in designing multiplex PCRs. The reduction in the number of oligonucleotides does not only reduce complexity of the reaction but also costs for consumables. We established a novel multiplex probe real-time PCR system, designated *LoopTag* [30], that is simple, robust, and incorporates melting curve analysis for the detection and differentiation of European species belonging to the *B. burgdorferi* s.l. complex.

## 2. Materials and Methods

### 2.1. qPCR Reaction and Melting Curve Analysis

Real-time PCRs were performed with a LightCycler® 1.5 and a LightCycler® 2.0 (Roche, Germany) using LightCycler® FastStart DNA Master HybProbe Kit (Roche, Germany). The PCR program encompasses 95 °C/7min initial denaturation followed by 45 cycles comprising 95 °C/4 s denaturation, 62 °C/25 s annealing, and 72 °C/15 s elongation. Afterward, a melting curve analysis was performed with 95 °C/3 s, 50 °C/10 s, 40 °C/20 s followed by a constant increase of 0.2 °C/s until 85 °C. The amplification was monitored during the annealing phase. The design of primers and probes was done following principles described in [31]. The formation of secondary structures and oligonucleotide dimers was studied with *Mfold* [32] and *PerlPrimer* [33], respectively. Primer and dye-labeled probes were purchased from Biotez (Germany) and IBA (Germany). The forward primer had the sequence 5${}^{\prime }$ATG GAG CCG CAA TCA TTG CCA TTG CAG A 3${}^{\prime }$ (GC: 50%, 28 nt, Tm${}_{in\;silico}$: 71.67 °C) which included the target-unspecific 5${}^{\prime }$-sequence (bold), and the *Borrelia*-specific primer sequence as published by Schwaiger et al. (2001) [29]. At its 5${}^{\prime }$-end it was labeled with fluorescein isothiocyanate (FITC, Emission: 510 nm). The reverse primer was non-labeled and had the *Borrelia*-specific sequence 5${}^{\prime }$AGC AAA TTT AGG TGC TTT CCA A3${}^{\prime }$ (GC: 36%, 22 nt, Tm${}_{in\;silico}$: 59.54 °C) as described by Schwaiger et al. (2001) [29].

The primer pair amplified a sequence of the flagellin gene with a size of 180 base pairs. The internal amplification control was synthesized by Biotez and had the sequence 5${}^{\prime }$GCA ATC ATT GCC ATT GCA GAG GCG GTT TGC GTA TTG GGC GCC AGG GTG GTT TTT CTT TTC ACC AGC GAG ACG GGC AAC AGC TGA TTG CCC TTC ACC GCC TGG CCC TGA GAG AGT TGC AGC AAG CGG TCC ACG CTG GTT GGA AAG CAC CTA AAT TTG C 3${}^{\prime }$. This probe design results in different melting temperatures for each gene (Table 1). The amplification control was amplified with the *Borrelia*-specific primer pair. For the detection and differentiation of *Borrelia* species a probe with the sequence 5${}^{\prime }$CAA TGA CAG ATG AGG TTG TAG CAG CAA CAA CTA ATA GTA GTG GCT CCA T 3${}^{\prime }$ was designed based on the alignment of the *Borrelia* flagellin gene. At its 3${}^{\prime }$-end it was labeled with Atto 590 (emission: 640 nm). The probe for the detection of the amplification control had the sequence 5${}^{\prime }$TGA AAA GAA AAA CCA CCC TGG CGC CCA AAG TGG CTC CAT 3${}^{\prime }$ and was 3${}^{\prime }$-labeled with Cy5.5 (emission: 705 nm). Color compensation was performed according to the manufacturer’s recommendations.

### 2.2. *Borrelia* Strains and DNA Extraction and qPCR Conditions

*Borrelia* species and strains are listed in Table 2. Genomic DNA was isolated using QIAamp DNA Mini Kit (Qiagen Corporation, Hilden, Germany). Genomic DNA was stored frozen in ddH${}_{2}$O until further use. For PCR, 5 μL template was mixed with 5 μL reaction mixture (8 mM MgCl${}_{2}$, 0.4 μM primer and probes, 8.24 × 10${}^{-12}$μM internal amplification control, 2× FastStart reaction mix). For determination of the detection limits (LoD) a dilution series ranging from 1 pg to 0.1 fg DNA per reaction was performed. The samples were provided semi-blinded by the German National Reference Center for *Borrelia* (Germany). Samples with unknown quantities of samples were provided for this purpose and were scanned using the *LoopTag* system. The Cq values and melting points determined by *LoopTag*-PCR were finally compared with the sample amounts. DNA concentrations were quantified with a NanoDrop instrument (Thermo Fisher, Waltham, MA, USA). At this point, it should be noted that the NanoDrop is not always the best nucleic acid quantification method [34,35,36]. An evaluation of further methods is recommended. The DNA was diluted in ddH${}_{2}$O. For comparing the *LoopTag* system with the intercalating dye EvaGreen [21,37] (Biotium, Fremont, CA, USA) one sample (*B. lusitaniae* Poti B3, 60 ng/μL) was diluted in water up to 1:10${}^{7}$, and the same primer pair was used. PCR tests were performed in triplicates for each dilution. After each PCR reaction melting curves were analyzed.

#### Data Analysis

Amplification data and melting curve data were exported from the LightCycler as comma separated values. *RKWard* [38] (v. 0.7.2) and dedicated *R* packages were used for all analysis as described here [39]. The *report* package (v. 0.3.0) [40] was used for report generation. In detail, amplification curves were preprocessed with functions from the *chipPCR* package [41] (v. 1.0.2). The RFU values are the ratio of the 640/530 channel. The Cq values were calculated by the scale-insensitive marker second-derivative maximum (SDM) [42]. The amplification efficiency was determined with the *effcalc* function from the *chipPCR* package using a decadic dilution series. Melting curves were analyzed using the *MBmca* package [43] (v. 1.0.1.1) as described in [39].

## 3. Results

### 3.1. Mechanism of the *LoopTag* System

**Figure 1:**
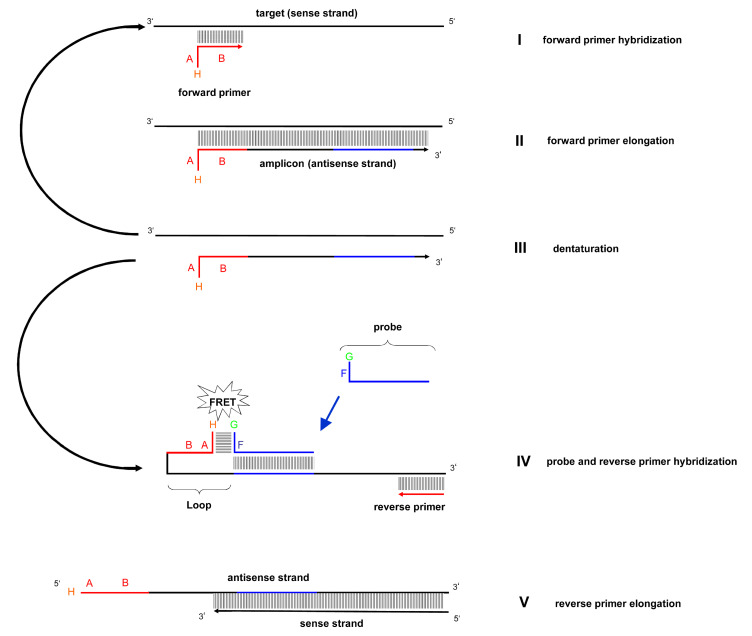
The diagram shows the successive stages of the LoopTag system during PCR. Please note the different reactions of the target, and the amplicon strand respectively. The mechanism is explained in the text. The forward primer of the *Borrelia* flagellin primer pair carries an 8 base pair long target-unspecific 5${}^{\prime }$-sequence (A), and a 20 base pair long target-specific sequence (B) which hybridizes to the target (stage I). The forward primer is elongated by the polymerase (stage II). After denaturation (stage III) the probe hybridizes to the antisense strand. The target-unspecific 5${}^{\prime }$-sequence of the antisense strand (A) is designed to form a loop by hybridization to its complementary sequence, which is the target-unspecific part (F) of the probe. The loop brings together the fluorescence donor (G) and the fluorescence acceptor (H), both covalently attached to the probe and the forward primer, respectively. This results in a FRET signal. The FRET signal is proportional to the number of amplification products. The reverse primer of the *Borrelia* flagellin primer pair hybridizes to the antisense strand (stage IV) and is elongated by the polymerase (stage V). After stage 5, the circle continues.

The principle of the *LoopTag* real-time PCR probe system is shown in Figure 1. The forward primer hybridizes onto a target 3${}^{\prime }$-5${}^{\prime }$ DNA strand leading to elongation during PCR. Denaturation of the newly formed strand leads to a new 5${}^{\prime }$-3${}^{\prime }$ strand with the attached primer plus acceptor dye. The binding of the probe and reverse primer hybridization leads to looping allowing the transmission of energy from a donor molecule to an acceptor molecule. The transfer of energy leads to a reduction in the donor’s fluorescence intensity and consequently its excited state lifetime, and to a corresponding measurable increase in the acceptor’s emission intensity that can be monitored during real-time PCR amplification. Reverse primer elongation then occurs leading to the formation of a new DNA strand (Figure 1).

### 3.2. Amplification of the Flagellin Gene

**Figure 2:**
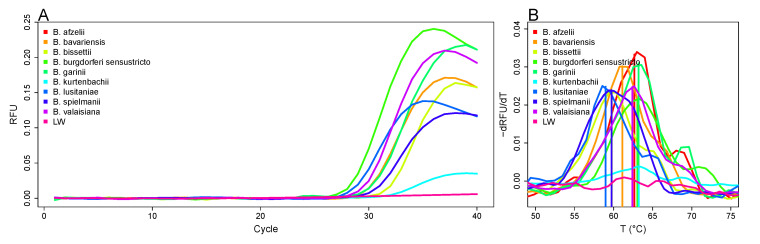
Amplification of the flagellin gene. (**A**) Plots of the amplification curves for the detection of the flagellin gene of various *Borrelia*. Flagellin gene of: *B. afzelii* PKo, *B. bavariensis* PBi, *B. bissettii* PGeb, *B. burgdorferi* s.s. B31, *B. garinii* PLa, *B. kurtenbachii* 25015, *B. lusitaniae* Poti B2, *B. spielmanii* PSig2, and *B. valaisiana* VS116. (**B**) Melting peak analysis. Single melting peaks of the *Borrelia* species: *B. afzelii* PKo (62.4 ± 0.1 °C), *B. bavariensis* PBi (61.1 ± 0.4 °C), *B. bissettii* PGeb (60.2 ± 0.8 °C), *B. burgdorferi* s.s. B31 (63.7 ± 0.7 °C), *B. garinii* PLa (62.3 ± 0.3 °C), *B. kurtenbachii* 25015 (63.6 ± 0.3 °C), *B. lusitaniae* Poti B2 (59.7 ± 1.0 °C), *B. spielmanii* PSig2 (59.1 ± 0.5 °C), and *B. valaisiana* VS116 (62.4 ± 0.2 °C). A non-template water control (LW) exhibited no melting peak. Melting temperatures were calculated on the basis of three independent experiments.

An amplification of the flagellin gene of the following species and strains of the *B. burgdorferi* s.l. complex was detected: *B. valaisiana* strain VS116; *B. lusitaniae* strains Poti B2 and Poti B3; *B. burgdorferi* s.s. strains B31, PKa2, and PBre; *B. spielmanii* strain PSig2; *B. bavariensis* strain PBi; B. garinii strains PLa PBr, PHei, TN, PRef, and PWudII; *B. afzelii* strains PKo, PGau, and PVPM; *B. bissettii* strain PGeb; *B. kurtenbachii* strain 25015. Regarding the relapsing fever group *Borreliae*, the species *B. miyamotoi* and *B. recurrentis* were not detectable while *B. parkerii*, *B. anserina*, *B. duttonii*, and *B. turicatae* were detectable at very high template concentration per reaction (more than 0.64 ng or 0.32 ng for *B. turicatae*, data not shown). Results of the amplification curves of several strains are shown in Figure 2A. Our system did not amplify the flagellin gene of related species like two *Treponema phagedenis* strains and two pathogenic *Leptospira* strains as well as two *Escherichia coli* (*E. coli*) isolates.

### 3.3. Differentiation of *Borrelia* Species Based on Melting Curve Analysis

The *LoopTag* system was established for the multiplex differentiation of pathogens. With one primer pair (low PCR complexity) the respective target is amplified, quantified, and defined by the melting temperature (Tm) of the PCR product. As shown in Figure 2B each PCR product of a specific flagellin sequence had a defined melting temperature. PCR products of all strains of one species had the same melting temperature (Table 1, Appendix A).

**Figure 3:**
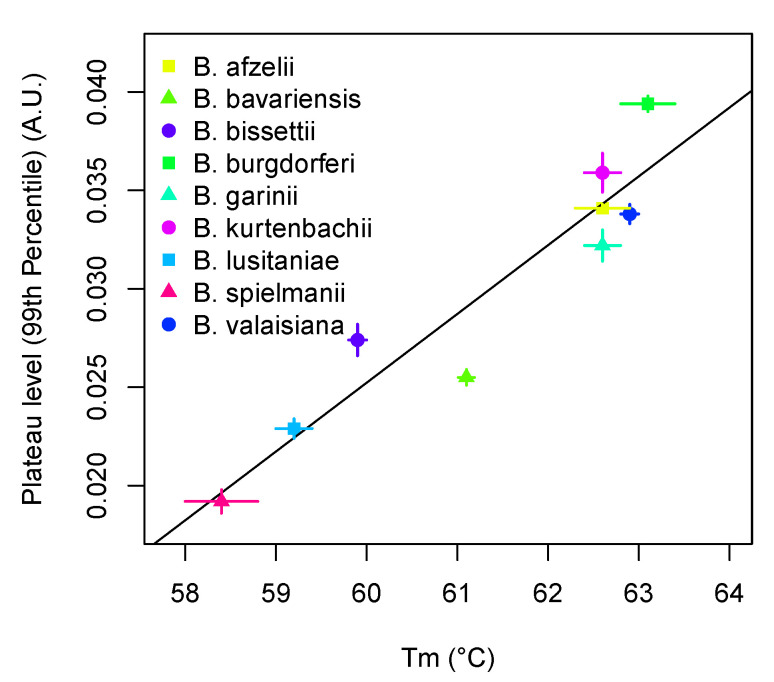
Probe affinity per species affects the plateau height.

The melting temperature is an affinity measure of the probe to the target sequence. Therefore, we analyzed the relationship between the probe affinity per species (melting temperature) on the plateau height. Since the melting temperatures between the species may differ greatly, we studied how the probe affinity per species affects the plateau height. For minimizing the influence of noise in amplification curves, not the maximum but the 99th percentile of the amplification curve was used as the plateau value (Figure 3). The Pearson’s product-moment correlation between the species-specific melting temperature and plateau height is significant, large, and positive (r(7) = 0.94, 95% CI (0.74, 0.99), *p* < 0.001).

### 3.4. Internal Control for the Detection and Differentiation of *Borrelia* Species

An internal control is a prerequisite for specific pathogen detection in clinical samples. We established a control system based on an artificial DNA sequence, which was framed by sequences complementary to the *Borrelia* flagellin primer. Thus, the internal control sequence was amplified with the same primers like the *Borrelia* target sequences. The probe of the internal control, bound to the artificial DNA sequence, was labeled with Cyanine 5.5 (Cy5.5). The *Borrelia*-specific probe was labeled with the fluorescence dye Atto 590 (Atto-Tec, Siegen, Germany). Conclusively, amplification of the internal control was quantifiable in a second non-interfering fluorescence channel.

### 3.5. Sensitivity and Efficiency

**Figure 4:**
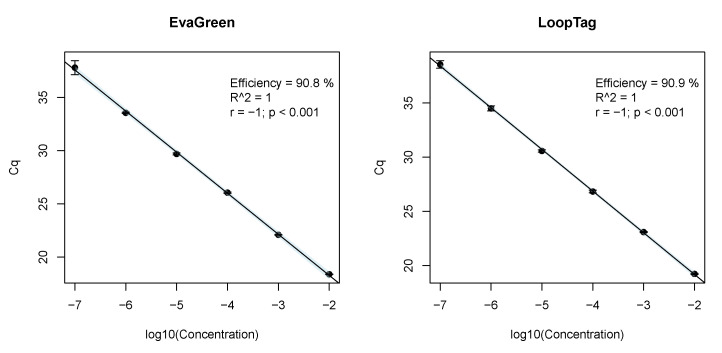
Comparison of the *LoopTag* system to a system using the common intercalating dye EvaGreen for detection. We found no pertinent differences between the *LoopTag* and EvaGreen detection systems. The amplification efficiency was approximately 91%.

The comparison of the *LoopTag* system to a system using EvaGreen detection (as intercalating dye) revealed a similar efficiency and sensitivity (Figure 4). The minimal detectable amount of genome equivalents per strain is listed in Table 2.

## 4. Discussion

Laboratory *Borrelia* diagnosis refers to some methods with known limitations. Antibody assays suffer from low sensitivity during an early stage of disease or from lower specificity caused by cross-reactions. Moreover, differentiation between an active infection and antibodies from former infections is difficult as antibodies persist after an infection has been overcome: in the normal population the prevalence of antibodies to *B. burgdorferi* is up to 25%, depending on age and gender. Additionally, detection by culture, combined with microscopic detection or serological testing, is in use [44]. However, the method is time-consuming, needs special equipment, special experience, and the sensitivity is low, ranging from 40–70% for skin biopsy specimens, and 10–30% for cerebrospinal fluid. Although false-positive or false-negative results occur, PCR is the most modern technique for detecting human pathogenic *Borrelia burgdorferi* (sensu lato) complex species [9,44,45]. PCR tests overcome some limitations of current diagnostic techniques and are valuable additional tools for the diagnosis of Lyme borreliosis. Real-time quantitive PCR has sped up the molecular diagnosis of pathogens during the last decade. This process was impelled essentially by the development of different molecular probe systems.

Here, we describe a probe system designed for a multiplex quantitative PCR test for the detection and differentiation of *Borrelia* species. In comparison to other probe systems, the most important advantage of this system is that only a single probe with a simple 3${}^{\prime }$ end fluorescent marking is required for the melt curve analysis. The second label, e.g., a standard fluorescein label, is conjugated to the 5${}^{\prime }$-end of one primer. This is advantageous for multiplexing as this approach reduces costs, and the number of oligonucleotides per reaction. The degree of multiplexing depends on the sensor technology of the measuring instrument, the available FRET pairs, the probe regions (differences in Tms), and the PCR conditions (e.g., salt concentration, pH value). We focused on a multiplex level that is sufficient for this diagnostic application.

Loop formation may initiate complex secondary DNA structures, since loops may also hybridize internally within minimally complementary regions. However, loop or hairpin formation is a molecular process often applied in probe systems [21,24]. In this respect, our *LoopTag* system is similar to Molecular Beacons, which also form a stem-loop-like structure only upon hybridization to a target. This approach allows for a multiplexed melting point analyzes [21,28]. The properties of the *LoopTag* system are that fewer probes/primers are in the mix, that the position of the detection probes can be flexibly selected and that the stem sequences are suitable for other target detections.

Loop formation of the *LoopTag* system between one of the primers, and the probe appears not to inhibit amplification. In contrast to other FRET probe systems reported [21], the *LoopTag* system shows large variability about the binding site of the probe. Nonetheless, the loop structure is crucial for melting curve analysis, which demands a careful amplicon design. The shape of the amplification curves regularly shows a hook-like shape, as is also known from other probe systems [46]. Each standard PCR, independent of the amplicon length, could be adjusted by adding the stem sequences to one primer and by designing the probe. This also applies to multiplex PCRs.

The *LoopTag* system utilizes sequence-specific probes in combination with the primers. It is well accepted that this combination has a higher specificity than intercalating dyes. The specificity of the applied test system is indeed very good. All included European species of the *B. burgdorferi* s.l complex were detectable. Relapsing fever *Borrelia* were not detectable or only at unphysiological high DNA concentrations. Small differences in the gene sequence of the flagellin gene within the species of the *B. burgdorferi* s.l complex results in different Tms. However, the high sequence homology of the flagellin gene and the standard deviations of the Tms makes it difficult to distinguish each species from another by several degrees Celsius. This is a natural limit for the multiplexing degree. Each system has defined technical limits, such as serological tests (see introduction). We have tested known strains and therefore cannot study the melting temperatures of all existing strains. Whenever the sequences have a high similarity, some strains will have similar melting temperatures. For example, *B. lusitaniae* and *B. bissettii* are similar in their melting temperature. The pathogenicity of both strains has not yet been fully clarified and they do not occur frequently. In an unknown sample with a melting temperature (e.g., 60 °C), pathogens would be detected due to the primer specificity but would not be differentiated. In such cases, information, such as epidemiological data, should be taken into account more strongly. Further analyses (e.g., sequencing, further LoopTag probe system) could also be carried out. Thus, our probe system can be used for the detection of borrelia to distinguish *B. burgdorferi* s.l. strains in the delimitation of relapse fever strains. The latter are detected by the present system in physiological concentrations. The differentiation between *B. burgdorferi* s.l. strains is possible with restrictions. Although individual species have very similar melting temperatures, it is possible to associate them with groups having the same melting temperature. The *LoopTag* system possesses a large linear detection range of at least between 10 and 1,000,000 copies per sample. Our system has not yet been tested in multiple global laboratories and therefore does not meet the requirements for a companion diagnostic (CDx). However, there are other application scenarios for our probe system. On the one hand, it can be used in clinical research to screen samples. On the other hand, the system can be used in routine clinical practice as a laboratory-developed test (LDT, use within a single laboratory), as we have disclosed all sequences in this study.

Another application is the use of the *LoopTag* system in combination with planar array technologies indicating its versatility. In a proof-of-concept study we transferred the *LoopTag* system for the multiplex detection of PCR products on the surface of microbeads for a real-time monitoring and surface melting curve analysis (see Appendix B).

## 5. Conclusions

We designed a new probe system, called *LoopTag*, for the detection and the differentiation of PCR products. In our study we applied the technology on *Borrelia* species. The system is simple and offers the ability to perform melting curve analysis. We validated the *LoopTag* system for the detection and differentiation of European species of the *B. burgdorferi* s.l. complex and the distinguishing from relapsing fever *Borrelia* species. We show a high specificity. We also show a high sensitivity down to 10 genome equivalents per PCR reaction.

## 6. Patents

The *LoopTag* system is patented [30].

## Figures and Tables

**Table 1:** Tms and their standard deviations per species. The melting points and the standard deviations were determined by means of 5–6 measurements.

Species	Melting Temperature (°C)
*B. afzelii*	62.4 ± 0.1
*B. bavariensis*	61.1 ± 0.4
*B. bissettii*	60.2 ± 0.8
*B. burgdorferii*	63.7 ± 0.7
*B. garinii*	62.3 ± 0.3
*B. kurtenbachii*	63.6 ± 0.3
*B. lusitaniae*	59.7 ± 1
*B. spielmanii*	59.1 ± 0.5
*B. valaisiana*	62.4 ± 0.2

**Table 2:** Minimal detectable amount of genome equivalents per species. DMAGE, Detectable minimal amount of genome equivalents per PCR reaction. *, data are based on determinations of in vitro cultivated *Borrelia* species. ${}^{§}$, different strains of one species. Serotypes: PHei, TN, PRef, PLa, PWudII.

*Borrelia*	Species	DMAGE
*Borrelia burgdorferi* s.l. complex	*B. afzelii*	≥10
	*B. bavariensis*	≥10
	*B. bissettii*	≥10
	*B. burgdorferi* s.s.	≥10
	*B. garinii*	PBr ${}^{§}$: approx. 10
		PHei: ≥10
		TN: approx. 700
		PRef: ≥10
		PLa: ≥10
		PWudII: ≥10
	*B. kurtenbachii*	100
	*B. lusitaniae*	≥10
	*B. spielmanii*	≥10
	*B. valaisiana*	≥10
Relapsing fever *Borrelia*	*B. anserina*	≥400.000 *
	*B. duttonii*	≥400.000 *
	*B. miyamotoi*	Not detectable
	*B. parkerii*	≥400.000 *
	*B. recurrentis*	Not detectable
	*B. turicatae*	≥200.000 *
Negative controls (other species)	*E. coli* (2 strains)	Not detectable
	*Leptospira* (2 strains)	Not detectable
	*Treponema phagedenis* (2 strains)	Not detectable

**Figure A1:**
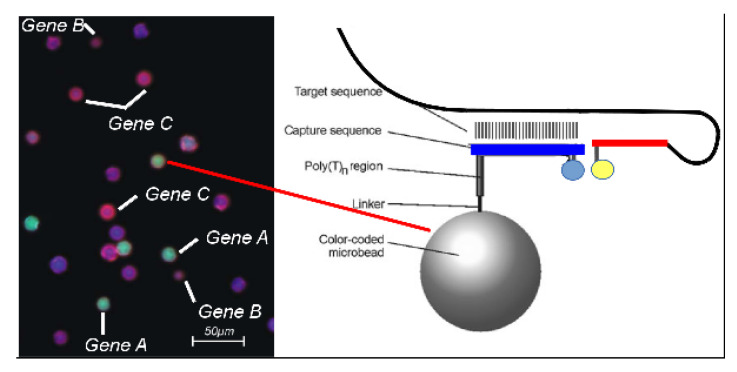
LoopTag probe system microbead real-time PCR. *LoopTag* formation. Right: Target specific *LoopTag* probes are assigned to a microbead population, which can be distinguished by its encoding dyes and size. In this cartoon three different populations encode the probe A (negative control), and the target sequences B (*stx1*) and C (*estA1*). Left: The target biomolecules are detected on the microbead surface as red corona. The *LoopTag* primer (red) labels the amplicon with a dye, in this case the quencher BHQ2 (yellow dot). One part of the *LoopTag* amplicon hybridizes to the microbead bound *LoopTag* capture probe (blue). The capture probe is labeled with a dye, in this case the fluorophor Atto647N (blue dot). The FRET signal is monitored in real-time during the annealing and elongation phase. Hence, hybridization analysis and differentiation by melting curve analysis are possible.

**Figure A2:**
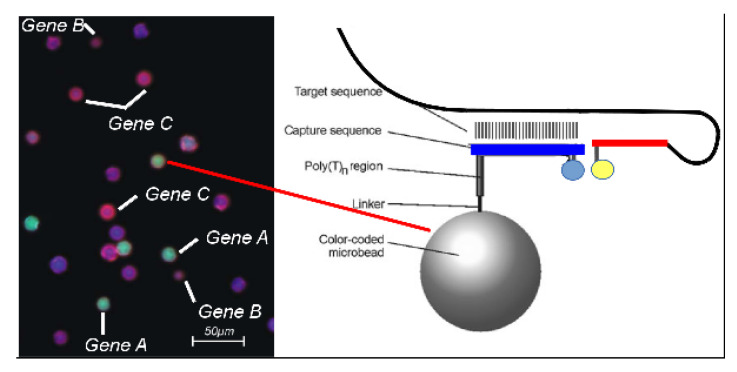
Quantitative PCR and melting curve analysis on the surface of microbeads. (**A**) Amplification of *E. coli* genes *stx1* nd *estA1* was monitored in a duplex *LoopTag* microbead assay. Negative controls (water control) were not amplified. (**B**) The melting temperature on the surface was determined for both genes. *stx1* and *estA1* had different melting temperatures of 63.2 °C and 53.9 °C, respectively.

## Data Availability

The data for this study are stored on https://codeberg.org/devSJR/LoopTag_data (accessed on 27 October 2021). This includes the raw data (file formats: txt, xlsx) of the amplification curves and melting curves from Roche’s LightCycler systems, and the VideoScan platform. All data were published under the GPL-3 or later license for research purposes.

## Abbreviations

   The following abbreviations are used in this manuscript:

A.U.	Arbitrary units
BHQ2	Black Hole Quencher 2
Cq	Cycle of quantification by SDM method
*estA1*	Heat-stable toxin I gene
FRET	Foerster Resonance Energy Transfer
qPCR	Quantitative PCR
RFU	Relative fluorescence units [Arbitrary Unit]
rMFI	Reference mean fluorescence value (see [47])
SDM	Second derivative maximum (see [42,48])
*stx1*	Shigatoxin 1 gene
Tm	Melting temperature

## Appendix A. Melting Point Analysis of Borrelia Specific Probes

**Table A1:** Pair-wise comparisons of the melting temperature of *Borrelia*. All combinations of melting points were subjected to the parametric pairwise multiple comparisons tests by means of the Tukey HSD test. All analyzes with a *p* value less than 0.05 are shown.

Comparison	Difference (°C)	Lower	Upper	*p* ${}_{\mathrm{adj}}$
*B. bavariensis* vs. *B. afzelii*	−1.55	−1.98	−1.12	<0.001
*B. bissettii* vs. *B. afzelii*	−2.70	−3.13	−2.27	<0.001
*B. burgdorferi* vs. *B. afzelii*	0.46	0.02	0.89	0.03
*B. lusitaniae* vs. *B. afzelii*	−3.48	−3.91	−3.04	<0.001
*B. spielmanii* vs. *B. afzelii*	−4.24	−4.67	−3.81	<0.001
*B. bissettii* vs. *B. bavariensis*	−1.15	−1.58	−0.72	<0.001
*B. burgdorferi* vs. *B. bavariensis*	2.00	1.57	2.44	<0.001
*B. garinii* vs. *B. bavariensis*	1.56	1.12	1.99	<0.001
*B. kurtenbachii* vs. *B. bavariensis*	1.52	1.09	1.96	<0.001
*B. lusitaniae* vs. *B. bavariensis*	−1.93	−2.36	−1.50	<0.001
*B. spielmanii* vs. *B. bavariensis*	−2.69	−3.12	−2.26	<0.001
*B. valaisiana* vs. *B. bavariensis*	1.79	1.35	2.22	<0.001
*B. burgdorferi* vs. *B. bissettii*	3.15	2.72	3.59	<0.001
*B. garinii* vs. *B. bissettii*	2.71	2.27	3.14	<0.001
*B. kurtenbachii* vs. *B. bissettii*	2.68	2.24	3.11	<0.001
*B. lusitaniae* vs. *B. bissettii*	−0.78	−1.21	−0.35	<0.001
*B. spielmanii* vs. *B. bissettii*	−1.54	−1.97	−1.11	<0.001
*B. valaisiana* vs. *B. bissettii*	2.94	2.50	3.37	<0.001
*B. garinii* vs. *B. burgdorferi*	−0.45	−0.88	−0.01	0.04
*B. kurtenbachii* vs. *B. burgdorferi*	−0.48	−0.91	−0.05	0.02
*B. lusitaniae* vs. *B. burgdorferi*	−3.93	−4.37	−3.50	<0.001
*B. spielmanii* vs. *B. burgdorferi*	−4.69	−5.13	−4.26	<0.001
*B. lusitaniae* vs. *B. garinii*	−3.49	−3.92	−3.05	<0.001
*B. spielmanii* vs. *B. garinii*	−4.25	−4.68	−3.81	<0.001
*B. lusitaniae* vs. *B. kurtenbachii*	−3.45	−3.89	−3.02	<0.001
*B. spielmanii* vs. *B. kurtenbachii*	−4.22	−4.65	−3.78	<0.001
*B. spielmanii* vs. *B. lusitaniae*	−0.76	−1.20	−0.33	<0.001
*B. valaisiana* vs. *B. lusitaniae*	3.71	3.28	4.15	<0.001
*B. valaisiana* vs. *B. spielmanii*	4.48	4.04	4.91	<0.001

## Appendix B. Duplex LoopTag qPCR on a Planar Microbead Assays

Aim: Planar microbead assays are an alternative to microarrays and suspension arrays since both enable the simultaneous monitoring of multiple targets in a single test. Different probe systems on microbead surfaces have been proposed [24]. We describe a qPCR approach based on microbeads, in which multiplexing is based on the number of microbead populations. The details on this coding principle are described in [24,47]. This is interesting for diagnostics, since assays with a high multiplex can in principle be built up. In our case study, an application of the *LoopTag* probe system is shown on a planar microbead assay. Figure A1 explains the concept of the *LoopTag* microbead probe system.

Methods and Materials: To keep this supplement concise we refer to a summary about our *VideoScan* platform in the study [47]. In short, it is a fully automated microscopy system that allows cells, solutions, microbead assays, or combinations thereof to be analyzed by means of bioimage informatics. Measurements can be carried out in qPCR format using the *VideoScan* platform.

In the *Borrelia* study we used *E. coli* as control. Since *Borrelia* sample material is precious, we decided to conduct the study with DNA material from *E. coli* from our strain collection. We picked two representative diagnostically relevant genes commonly associated with Shigatoxin-producing *E. coli* (STEC; Shigatoxin 1 gene *stx1*) and enterotoxigenic *E. coli* (ETEC; heat-stable toxin I gene *estA1*).

- Base primer sequences were taken from [49] and transformed into a *LoopTag* probe system as described above. An important difference is that a part of the probes are bound to the surface of microbeads, so that they can function as catcher molecules (Table A2). The gene *estA1* (length of amplification product: 158 bp) and *stx1* (length of amplification product: 211 bp) were used in this proof-of-concept study.
- Colonies of *E. coli* strains containing genes *estA1* and *stx1* were inoculated in 1.5 mL LB medium. After incubation at 37 °C overnight cells were separated, re-suspended in 300 μL of water, and lysed by heating at 99 °C for 10 min. Cell debris were removed by centrifugation and supernatant was stored at −20 °C. Thawed lysates were directly used as templates for PCR.

**Table A2:** Primer and probes for the microbead assay. Labels: Atto647N; BHQ2, Black Hole Quencher 2; 2B, dual biotin. Melting temperatures (Tm) were calculated with PerlPrimer.

Function	Sequence
*estA1* fw	BHQ2-ATCTACCAACTGAATCACTTGACTCTT (GC: 37%, 27 nt, Tm: 62.45 °C)
*estA1* rv	TTAATAACATCCAGCACAGG (GC: 40%, 20 nt, Tm: 55.57 °C)
*estA1* probe	2B-AGTCTCTAATGTAATTTTCTCTTTTGGTAGAT-Atto647N (GC: 28%, 32 nt, Tm: 61.51 °C)
*stx1* fw	BHQ2-ATGTATGTTGCAGGGATCAGTCGT (GC: 45%, 24 nt, Tm: 64.57 °C)
*stx1* rv	AGAACGCCCACTGAGATCATC (GC: 52%, 21 nt, Tm: 62.41 °C)
*stx1* probe	2B-GTCAACGAATGGCGATTTATCTGCATCCCGTACAT-Atto647N (GC: 45%, 35 nt, Tm: 62.41 °C)

Carboxylated PMMA microbeads were coated with streptavidin as described in [47] and subjected to a qPCR reaction and melting curve analysis in the VideoScan platform.

1. Briefly, 300,000 microbeads were re-suspended in 100 μL of 100 mM methylimidazole (MeIm, Sigma, Milwaukee, WI, USA) buffer (pH 7.0) containing 25 mg/mL *N*-(3-dimethy-laminopropyl)-*N*${}^{\prime }$-ethylcarbodiimide hydrochloride (EDC, Sigma). Activated microbeads were incubated with streptavidin solution (300 μg/mL in MeIm-buffer for 5 h at 50 °C) with continuous agitation for covalent cross-linking. After washing three times with TBST buffer, streptavidin-coated microbeads were ready for loading with biotinylated oligonucleotides. Microbeads were mixed with 100 μL TBST buffer containing 100 nM of 5${}^{\prime }$-bis-biotinylated and 3${}^{\prime }$-Atto647N-labeled oligonucleotides (biomers.net, Ulm, Germany). After 15 min incubation at room temperature, unbound oligonucleotides were removed by washing microbeads three times with 200 μL TBST. Finally, microbeads were exposed to 95 °C for 10 min.
2. All PCR reactions were conducted within a volume of 20 μL Biotherm polymerase buffer (Genecraft, Colone, Germany) containing 5 mM MgCl${}_{2}$, 200 μM of each dNTP, 250 μM of BHQ2 labeled fw primer, 250 μM of rv primer, 20–200 streptavidin-coupled microbeads with bound bis-biotinylated capture probes, 1 U Biotherm DNA-polymerase (Genecraft), and 1 μL of *E. coli* lysate as template. The mixture was transferred into a cavity of a nucleolink TopYield strip (Nunc, Roskilde, Denmark), covered with 30 μL of mineral oil, and the strip was placed into sockets of the heating and cooling unit. Here, a PCR was performed using the following program: 4 min, 94 °C, 40× (60 s 94 °C, 60 s 55 °C, 90 s 72 °C), 5 min 72 °C. We monitored microbead surface fluorescence while the temperature was increased at 1 °C/min starting from 35 °C to 90 °C. By use of the VideoScan technology fluorescence images of microbeads on the bottom of the well were taken after every cycle (55 °C). Images were evaluated with imaging processing software allowing the recognition of microbeads, the assignment to a population and the determination of their surface fluorescence.
3. The mean fluorescence value for every population (rMFI → RFU) was plotted against the cycle number leading to the shown real-time kinetic curve. The amplification curve data were pre-processed and analyzed using the *qpcR* (v. 1.4.1) [50], *MBmca*, and *chipPCR* packages. After finishing real-time duplex PCR, a melting curve analysis was done as described in [39,43].

Results and Conclusions:

We observed a curve shape, which resembles the typical sigmoid shape of quantitative PCRs (Figure 2). Amplification curves were fitted with a Richardson function. The Cq values were determined by the second derivative maximum method. The *stx1* amplification curve had a Cq value of 20.4, and the *estA1* amplification curve had a Cq value of 18.6 (Figure A2A). Next we performed a melting curve analysis on the surfaces of microbeads. The curve for *stx1* had a Tm value of 63.2 °C and for *estA1* a Tm value of 53.9 °C (Figure A2B). Negative controls were not amplified. In this proof-of-concept study the *LoopTag* probe system was easily applicable to a planar microbead-based assay.

Our measuring system was not further optimized for measuring the *LoopTag* system on microbeads. For example, we can observe a periodicity (wave-shaped amplification curve) as was also observed for commercial systems [51]. Though the signal-to-noise ratio is not optimal, this also led to the signal differing significantly from the background.

The evaluation and analysis was carried out in our case using the VideoScan platform. The principle should also be applicable to other planar assays. For the analysis of the image data, we use the proprietary *FastFluoScan* software (Attomol GmbH, Germany). However, other open source bioimage informatics software (as reviewed in [52]) could also be used for such scientific questions.
